# Supplementary material for: Ultrafast endocytosis at Caenorhabditis elegans neuromuscular junctions
Source: eLife. 2013 Sep 3;2:e00723. doi: 10.7554/eLife.00723 (PMC3762212; doi:10.7554/eLife.00723)
Supplement: Figure 2—source data 1. — DOI: http://dx.doi.org/10.7554/eLife.00723.005 [file elife00723s001.docx]

| Figure 2H and 2J: the numbers of docked and tethered vesicles in each profile were normalized by the area of active zones. | | | | | |
| --- | --- | --- | --- | --- | --- |
|  | Non-stimulated | | Stimulated (20 ms) | |  |
|  | N=102 profiles | | N=50 profiles | |  |
|  | Mean | SEM | Mean | SEM | P value |
| docked vesicles in active zone/profile | 2.5 | 0.1 | 0.8 | 0.1 | <0.0001 |
| tethered vesicles in active zone/profile | 3.1 | 0.2 | 3.0 | 0.3 | 0.5 |
