# Supplementary material for: Ultrafast endocytosis at Caenorhabditis elegans neuromuscular junctions
Source: eLife. 2013 Sep 3;2:e00723. doi: 10.7554/eLife.00723 (PMC3762212; doi:10.7554/eLife.00723)
Supplement: Figure 3—source data 1. — DOI: http://dx.doi.org/10.7554/eLife.00723.008 [file elife00723s002.docx]

| Figure 3D, E, and F: the numbers of docked and tethered vesicles in each profile were normalized by the area of active zones or perisynaptic zone | | | | | |
| --- | --- | --- | --- | --- | --- |
|  | Non-stimulated | | Stimulated (50 ms) | |  |
|  | N=102 | | N=114 | |  |
|  | Mean | SEM | Mean | SEM | P value |
| docked vesicles in active zone/profile | 2.5 | 0.1 | 1 | 0.1 | <0.0001 |
| docked vesicles in perisynaptic zone/profile | 0.11 | 0.04 | 0.10 | 0.04 | 0.95 |
| tethered vesicles in active zone/profile | 3.1 | 0.2 | 2.6 | 0.2 | 0.21 |
