# Supplementary material for: Ultrafast endocytosis at Caenorhabditis elegans neuromuscular junctions
Source: eLife. 2013 Sep 3;2:e00723. doi: 10.7554/eLife.00723 (PMC3762212; doi:10.7554/eLife.00723)
Supplement: Figure 4—source data 1. — DOI: http://dx.doi.org/10.7554/eLife.00723.011 [file elife00723s003.docx]

| Figure 4D, E, and F: the numbers of docked and tethered vesicles in each profile were normalized by the area of profiles. | | | | | |
| --- | --- | --- | --- | --- | --- |
|  | Non-stimulated | | Stimulated (no retinal) | |  |
|  | N=102 profiles | | N=51 profiles | |  |
|  | Mean | SEM | Mean | SEM | P value |
| total docked vesicles /profile | 2.6 | 0.1 | 2.7 | 0.1 | 0.9 |
| total vesicles/profile | 28 | 1 | 26 | 1 | 0.55 |
| total tethered vesicles/profile | 3.5 | 0.3 | 3.4 | 0.5 | 0.82 |
|  | Non-stimulated | | Stimulated (20 ms) | |  |
|  | N=102 profiles | | N=50 profiles | |  |
|  | Mean | SEM | Mean | SEM | P value |
| total docked vesicles /profile | 2.6 | 0.1 | 0.9 | 0.1 | <0.0001 |
| total vesicles/profile | 28 | 1.0 | 23 | 1.2 | 0.003 |
| total tethered vesicles/profile | 3.5 | 0.3 | 3.7 | 0.3 | 0.41 |
|  | Non-stimulated | | Stimulated (50 ms) | |  |
|  | N=102 profiles | | N=114 profiles | |  |
|  | Mean | SEM | Mean | SEM | P value |
| total docked vesicles /profile | 2.6 | 0.1 | 0.9 | 0.1 | <0.0001 |
| total vesicles/profile | 28 | 1.0 | 23 | 0.9 | 0.002 |
| total tethered vesicles/profile | 3.5 | 0.3 | 2.8 | 0.2 | 0.06 |
|  | Non-stimulated | | Stimulated (100 ms) | |  |
|  | N=102 profiles | | N=83 profiles | |  |
|  | Mean | SEM | Mean | SEM | P value |
| total docked vesicles /profile | 2.6 | 0.1 | 1 | 0.1 | <0.0001 |
| total vesicles/profile | 28 | 1.0 | 22 | 1 | 0.001 |
| total tethered vesicles/profile | 3.5 | 0.3 | 2.7 | 0.3 | 0.07 |
|  | Non-stimulated | | Stimulated (300 ms) | |  |
|  | N=102 profiles | | N=89 profiles | |  |
|  | Mean | SEM | Mean | SEM | P value |
| total docked vesicles /profile | 2.6 | 0.1 | 0.9 | 0.1 | <0.0001 |
| total vesicles/profile | 28 | 1.0 | 26 | 1.2 | 0.63 |
| total tethered vesicles/profile | 3.5 | 0.3 | 3.7 | 0.4 | 0.69 |
|  | Non-stimulated | | Stimulated (1 s) | |  |
|  | N=102 profiles | | N=111 profiles | |  |
|  | Mean | SEM | Mean | SEM | P value |
| total docked vesicles /profile | 2.6 | 0.1 | 1.3 | 0.1 | <0.0001 |
| total vesicles/profile | 28 | 1.0 | 25 | 1.1 | 0.63 |
| total tethered vesicles/profile | 3.5 | 0.3 | 4.4 | 0.3 | 0.05 |
|  | Non-stimulated | | Stimulated (3 s) | |  |
|  | N=102 profiles | | N=91 profiles | |  |
|  | Mean | SEM | Mean | SEM | P value |
| total docked vesicles /profile | 2.6 | 0.1 | 2.4 | 0.2 | 0.97 |
| total vesicles/profile | 28 | 1.0 | 26 | 1.0 | 0.65 |
| total tethered vesicles/profile | 3.5 | 0.3 | 3.6 | 0.4 | 0.84 |
|  | Non-stimulated | | Stimulated (10 s) | |  |
|  | N=102 profiles | | N=121 profiles | |  |
|  | Mean | SEM | Mean | SEM | P value |
| total docked vesicles /profile | 2.6 | 0.1 | 2.6 | 0.1 | 0.98 |
| total vesicles/profile | 28 | 1.0 | 27 | 0.8 | 0.8 |
| total tethered vesicles/profile | 3.5 | 0.3 | 3.4 | 0.2 | 0.77 |
