# Supplementary material for: Ultrafast endocytosis at Caenorhabditis elegans neuromuscular junctions
Source: eLife. 2013 Sep 3;2:e00723. doi: 10.7554/eLife.00723 (PMC3762212; doi:10.7554/eLife.00723)
Supplement: Figure 6—source data 1. — DOI: http://dx.doi.org/10.7554/eLife.00723.017 [file elife00723s005.docx]

| Figure 6E and F | | | | | |
| --- | --- | --- | --- | --- | --- |
|  | Non-stimulated | | Stimulated (no retinal) | |  |
|  | N=26 synapses | | N=12 synapses | |  |
|  | Mean | SEM | Mean | SEM | P value |
| number pits at adherens junctions (AJ) | 0.1 | 0.1 | 0.1 | 0.1 | ∅ |
| number large vesicles within 50 nm of AJ | 0.1 | 0.1 | 0.1 | 0.1 | ∅ |
|  | Non-stimulated | | Stimulated (20 ms) | |  |
|  | N=26 synapses | | N=12 synapses | |  |
|  | Mean | SEM | Mean | SEM | P value |
| number pits at adherens junctions (AJ) | 0.1 | 0.1 | 0.1 | 0.1 | ∅ |
| number large vesicles within 50 nm of AJ | 0.1 | 0.1 | 0.2 | 0.1 | 0.5 |
|  | Non-stimulated | | Stimulated (50 ms) | |  |
|  | N=26 synapses | | N=24 synapses | |  |
|  | Mean | SEM | Mean | SEM | P value |
| number pits at adherens junctions (AJ) | 0.1 | 0.1 | 0.2 | 0.1 | 0.5 |
| number large vesicles within 50 nm of AJ | 0.1 | 0.1 | 0.2 | 0.1 | 0.5 |
|  | Non-stimulated | | Stimulated (100 ms) | |  |
|  | N=26 synapses | | N=19 synapses | |  |
|  | Mean | SEM | Mean | SEM | P value |
| number pits at adherens junctions (AJ) | 0.1 | 0.1 | 0.6 | 0.2 | <0.05 |
| number large vesicles within 50 nm of AJ | 0.1 | 0.1 | 0.2 | 0.1 | 0.5 |
|  | Non-stimulated | | Stimulated (300 ms) | |  |
|  | N=26 synapses | | N=20 synapses | |  |
|  | Mean | SEM | Mean | SEM | P value |
| number pits at adherens junctions (AJ) | 0.1 | 0.1 | 1.45 | 0.4 | <0.001 |
| number large vesicles within 50 nm of AJ | 0.1 | 0.1 | 0.2 | 0.1 | 0.5 |
|  | Non-stimulated | | Stimulated (1 s) | |  |
|  | N=26 synapses | | N=23 synapses | |  |
|  | Mean | SEM | Mean | SEM | P value |
| number pits at adherens junctions (AJ) | 0.1 | 0.1 | 0.9 | 0.2 | <0.001 |
| number large vesicles within 50 nm of AJ | 0.1 | 0.1 | 0.4 | 0.1 | <0.05 |
|  | Non-stimulated | | Stimulated (3 s) | |  |
|  | N=26 synapses | | N=19 synapses | |  |
|  | Mean | SEM | Mean | SEM | P value |
| number pits at adherens junctions (AJ) | 0.1 | 0.1 | 0.5 | 0.2 | 0.06 |
| number large vesicles within 50 nm of AJ | 0.1 | 0.1 | 0.6 | 0.1 | <0.01 |
|  | Non-stimulated | | Stimulated (10 s) | |  |
|  | N=26 synapses | | N=26 synapses | |  |
|  | Mean | SEM | Mean | SEM | P value |
| number pits at adherens junctions (AJ) | 0.1 | 0.1 | 0.3 | 0.1 | 0.2 |
| number large vesicles within 50 nm of AJ | 0.1 | 0.1 | 0.1 | 0.1 | ∅ |
|  | | | | | |
| unc-13(s69);P*unc-17*::ChIEF | Non-stimulated | | Stimulated (50 ms) | |  |
|  | N=11 synapses | | N=16 synapses | |  |
|  | Mean | SEM | Mean | SEM | P value |
| number pits at adherens junctions (AJ) | 0 | 0 | 0 | 0 | ∅ |
| number large vesicles within 50 nm of AJ | 0 | 0 | 0 | 0 | ∅ |
| unc-13(s69);P*unc-17*::ChIEF | Non-stimulated | | Stimulated (1 s) | |  |
|  | N=11 synapses | | N=16 synapses | |  |
|  | Mean | SEM | Mean | SEM | P value |
| number pits at adherens junctions (AJ) | 0 | 0 | 0 | 0 | ∅ |
| number large vesicles within 50 nm of AJ | 0 | 0 | 0 | 0 | ∅ |
