# Supplementary material for: Ultrafast endocytosis at Caenorhabditis elegans neuromuscular junctions
Source: eLife. 2013 Sep 3;2:e00723. doi: 10.7554/eLife.00723 (PMC3762212; doi:10.7554/eLife.00723)
Supplement: Figure 7—source data 1. — DOI: http://dx.doi.org/10.7554/eLife.00723.020 [file elife00723s006.docx]

| Figure 7D Number of large vesicles | | | | |
| --- | --- | --- | --- | --- |
| Non-stimulated | | Stimulated (1s) | |  |
| N=10 synapses | | N=7 synapses | |  |
| Mean | SEM | Mean | SEM | P value |
| 2.7 | 0.4 | 2.7 | 0.6 | 0.92 |
|  |  |  |  |  |
| Figure 7E Diameter of large vesicles | | | | |
| Non-stimulated | | Stimulated (1s) | |  |
| N=10 synapses | | N=7 synapses | |  |
| Mean | SEM | Mean | SEM | P value |
| 59.0 | 5.9 | 85.7 | 8.5 | 0.01 |
